# Rectus Abdominis Repair Compared to Posterior Wall Mesh Reinforcement in Athletes With Sportsman’s Hernia: A Systematic Review

**DOI:** 10.7759/cureus.78580

**Published:** 2025-02-05

**Authors:** Jonathan McKeeman, Benjamin Johnson, Jessica C Rivera

**Affiliations:** 1 Orthopedic Surgery, St. Luke's Hospital, Bethlehem, USA; 2 Internal Medicine, University of South Florida, Tampa, USA; 3 Orthopaedic Surgery, Louisiana State University Health Sciences Center, New Orleans, USA

**Keywords:** athletic pubalgia, core muscle injury, gilmore’s groin, lower abdominal pain, sports hernia

## Abstract

Athletic pubalgia is a relatively uncommon injury that is not fully understood. There are few high-level studies comparing treatments of athletic pubalgia, and this investigation seeks to utilize original articles to compare two common techniques for treatment of athletic pubalgia. The purpose of this study is to compare two prominent procedures, i.e., rectus abdominus repair and posterior wall reinforcement, through a systematic review. Inclusion/exclusion criteria were identified and then applied to search strategies in PubMed and MEDLINE. Two reviewers screened articles based on the agreed-upon criteria in a primary screen of titles and abstracts and a secondary screen of full-text articles. A total of 59 full-text articles were reviewed, and 13 were selected for this study. The study designs included seven prospective cohort studies, five retrospective cohort studies, and one randomized control trial. There were five studies with rectus abdominus repair intervention and eight studies with posterior wall reinforcement intervention. The median age range was 22-32. The mean follow-up time ranged from one month to 12.5 years. The success rate ranged from 76% to 96% for rectus abdominus repair and from 72% to 98% for posterior wall reinforcement. The two procedures performed about the same as the success rate for both procedures ranged from about 70% to 90%. The lack of standardization in clinical outcomes makes comparison across studies difficult. It is hard to discern which procedure actually performs better, and thus future research in this area needs to be conducted to focus on more specific outcomes, frequent follow-up, and standardization of outcome measures.

## Introduction and background

Athletic pubalgia or “sportman's hernia” is a relatively uncommon injury although it occurs most frequently in the athletic population, hence the use of “sport” to describe what is not considered to be a true hernia. The prevalence of groin pain in the athletic population is estimated at around 5-10% [1]. Although uncommon, it accounts for approximately 5-6% of career-long injuries and is potentially a career-ending injury [2]. There is also not a true consensus on the pathology involved in athletic pubalgia, but it is believed to be an injury to one or more pubic structures, which include the pubic symphysis enthesis, the inguinal canal, the rectus abdominus, the conjoint tendon or myotendinous junction, and the adductor attachment site [2]. Making the pathology even more difficult to discern is the lengthy differential, which includes hip pathology, such as femoroacetabular impingement and arthritis, referred pain from spinal pathology, and testicular pathology including true hernias. In addition, some of the conditions on the differential list have a high comorbidity with athletic pubalgia, so it is necessary to thoroughly examine a patient presenting with groin pain. However, although the differential list is long, once many of the other pathologies have been excluded, athletic pubalgia is the accepted diagnosis. This is described as a “functional pain syndrome caused by overuse of the different elements composing the pubis: the oblique and rectus abdominis muscles, the osseous pubic rami and the adductor muscle” [3]. Oftentimes, imaging on plain X-ray, bone scan, ultrasound, and magnetic resonance imaging (MRI) is negative, so athletic pubalgia remains a clinical diagnosis of exclusion, but MRI is the standard for musculoskeletal pelvic imaging although a specific lesion is not often found. However, Zoga et al. asserted that MRI can provide specific findings in athletic pubalgia with the appropriate technique, which they believe involves analyzing musculoskeletal groin pain on a continuum of injury termed “core muscle injury” [4].

Conservative treatment is the first line for athletic pubalgia and includes rest, ice, non-steroidal anti-inflammatories, formal physical therapy, and local injection [1]. Many patients (around 80%) [5] do well with conservative treatment; when conservative management fails, surgical options are explored, and currently, there is no consensus on the preferred surgical treatment. There are various options of surgical treatment that have been attempted depending on the suspected nature of the injury, which include hernia repair, tenotomies of muscle attachments near the pubic bone, neurolysis of nerves perceived to be causing the pain, and reattachment of supposed torn structures such as the rectus abdominus [6,7]. Seemingly regardless of the surgical treatment chosen, results are “good” or “excellent” in 60-80% of cases [7].

A systematic review in 2008 analyzed the literature surrounding athletic pubalgia and sought to give a greater understanding of the prevalent ideas in the nature of the pathology of the injury and explain the rationale behind the targeted conservative and surgical treatments [8]. The purpose of this systematic review is to narrowly focus on the comparison of two of the predominant surgical interventions of athletic pubalgia in young athletes (under 40), namely, rectus abdominus repair and posterior wall mesh reinforcement. The outcomes used for the comparison of mean follow-up time include postoperative pain in terms of which month the patient no longer felt pain or Visual Analog Scale (VAS) pain score in the absence of a month-to-month timeline, return to sport time, and success rate as defined by Meyers et al. [6]. The success rate is defined as the percentage of athletes that achieved moderately successful or better results after surgery (see Table 1 for criteria).

**Table 1:** Description of success rate Success rate as defined by Meyers et al. [6]

Success Rate	Description
Highly successful	No pain and full return to sport at level or better
Moderately successful	Some pain, but full return to sport at level or better
Minimally successful	Improvement, but not to the preoperative level
Unsuccessful	No or minimal improvement

## Review

Inclusion and exclusion criteria

Eligible studies for review included all original articles on human patients of the athletic population with athletic pubalgia who underwent either posterior wall reinforcement with or without adductor tenotomy or rectus abdominus reattachment with or without adductor tenotomy. Excluded articles included basic science, animal, and cadaver studies. Articles were not restricted to patient demographics, except that patients must be part of the athlete population, which tends to be younger (median age below 40) and male due to the nature of the injury. Review articles, systematic review articles, book chapters, meta-analyses, and case reports were also excluded from this systematic review. Eligible articles included randomized control trials, prospective and retrospective cohort, case-control, and case series. Due to the high comorbidity of athletic pubalgia with conditions such as true hernia, low back pain, and femoroacetabular impingement, studies pertaining to primary inguinal hernia repair, discectomies, and femoroacetabular impingement syndrome treatment were excluded. The studies included must report at least one outcome for pain either via pain scale or time to no pain after surgery and studies included must also report return to sport time. The other clinical outcome, “success rate,” was determined from clinical outcomes regarding pain and return to sport. There were no limitations on publication date range or duration and articles not in English were excluded.

Search strategy

PubMed (National Library of Medicine) and MEDLINE (Ovid) were searched with the following search criteria: (Athletes AND Sports Hernia OR Core Muscle Injury OR Athletic Pubalgia OR Gilmore’s groin OR lower abdominal pain AND surgery AND Pain NOT Disc NOT Femoroacetabular impingement). Search criteria and bibliographies were stored in a Microsoft Excel workbook (Microsoft Corporation, Redmond, WA, USA) designed for systematic reviews. From the Excel workbook, duplicate articles were screened out. Then, search criteria and abstracts selected for screening were managed in Microsoft Excel.

Screening procedures

Two authors, both medical students, independently screened all titles and abstracts for exclusion criteria in Microsoft Excel. The screener agreement was considered substantial (92% agreement with a Cohen’s kappa = 0.66). Disagreement regarding study inclusion for full-text review was reconciled favoring full-text review on all but one article, which was excluded.

Full-Text Review Procedures

Each full-text article included in the screening procedure (59) was fully reviewed by two authors. Each study was analyzed for citation, study level, treatment, and outcome variables in line with the prescribed inclusion criteria. Studies were excluded based on previously described exclusion criteria and selected for inclusion based on whether the study fulfilled those requirements, including adequate measures for pain and return to sport time. Many studies were excluded due to a lack of clinical pain outcomes, and several more were excluded due to a lack of return to sports time. There were also a few articles excluded because they did not fit well within the two targeted procedures although they did treat the patient with surgery for athletic pubalgia. Finally, a few case series were excluded due to a lack of substantial evidence from the limited number of patients (less than 10). There was perfect agreement from the screeners (100% agreement with a Cohen’s Kappa = 1.0) although the screeners did defer on specific exclusion criteria on some articles.

**Figure 1:**
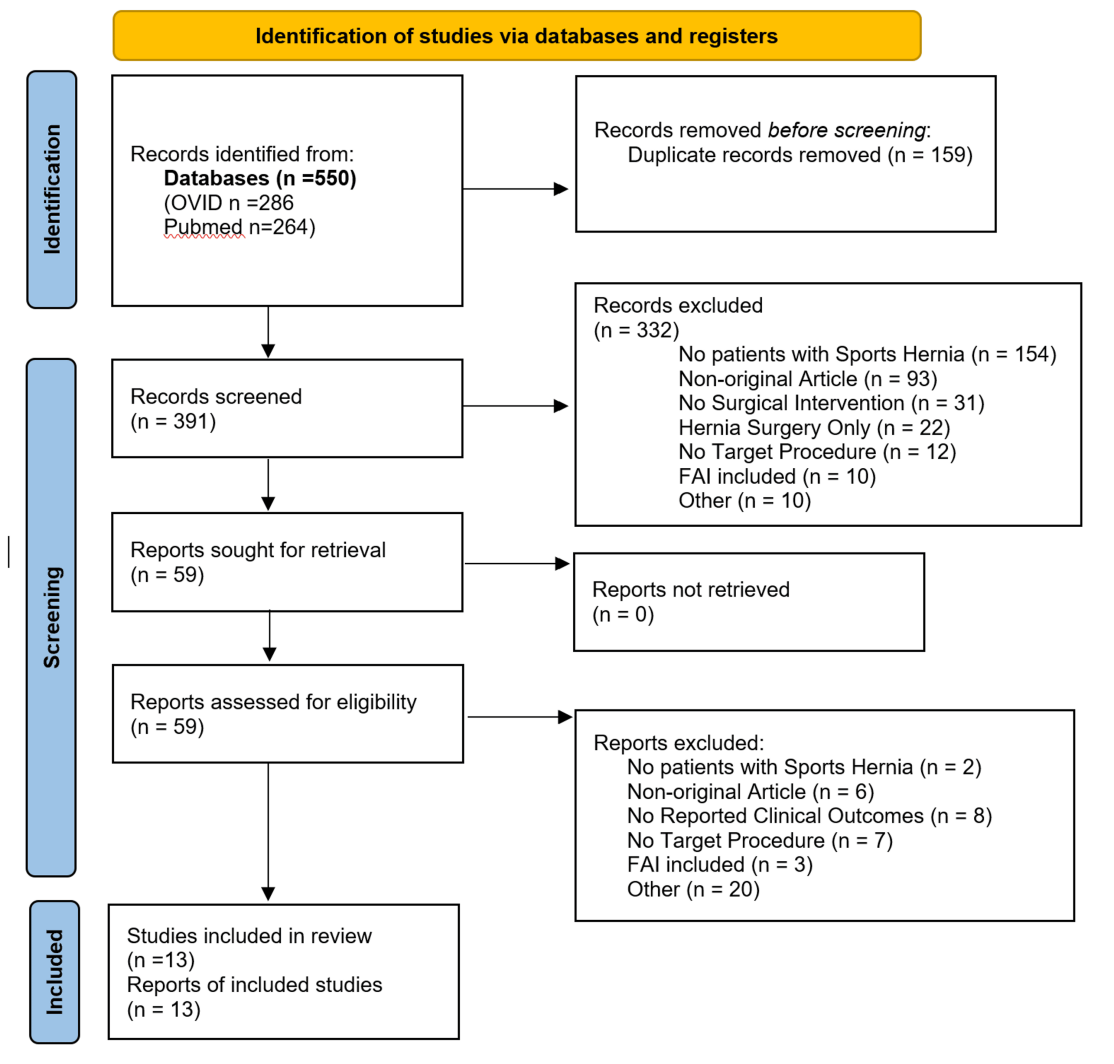
PRISMA flowchart detailing the systematic review process PRISMA: Preferred Reporting Items for Systematic Reviews and Meta-Analyses

The PubMed and OVID searches yielded 391 unique titles and abstracts to be screened. Of the 391, 332 titles and abstracts were excluded as detailed in the PRISMA chart. Fifty-nine full-text articles were reviewed and 46 were excluded with the majority falling into the “other” category. This category was reserved for more specific rule in/out criteria such as whether the study provided adequate clinical outcomes to calculate the “success rate.” Moreover, falling into the other category was whether the clinical outcomes such as pain score would reasonably allow comparison across studies. For example, articles only including pain outcomes immediately following surgery were not included due to a lack of follow-up with clinical outcomes. Lastly, articles deemed too small to provide substantial evidence for comparison such as case series fell under the category of other. There was a total of 13 studies included in this systematic review as seen in Figure 1 [9].

As seen in Table 2, the studies included had a median age range of 22-32, which incorporated the “young” criterion well [2,3,6,7,10-18]. All patients in the studies were athletes at various levels of competition ranging from recreational to professional. The study designs included seven prospective cohort studies, five retrospective cohort studies, and one randomized control trial. There were five studies with rectus abdominus repair intervention and eight studies with posterior wall reinforcement intervention. The posterior wall reinforcement was subdivided into totally extraperitoneal and Bassini repair. The mean follow-up time ranged from one month to 12.5 years. All studies provided outcomes for postoperative pain and return to sport time. One study used the VAS pain scale rather than “time to no pain” as the pain outcome. From the pain and return to sport outcomes, the success rate as defined by Meyer et al. was determined [6]. The success rate ranged from 76-96% for rectus abdominus repair and 72-98% for posterior wall reinforcement.

**Table 2:** Systematic review of the clinical outcome results for each study

References	Sample size	Subject mean age	Study design	Procedure	Mean follow-up time	Postoperative pain	Return to sport (time)	Success rate
Meyers et al. [6]	157	N/A	Prospective cohort	Rectus abdominus repair	3.9 years	89% no pain at six months	Three months (88%) 6 months (96%)	95%
									Biedert et al. [10]	24	25.8	Retrospective cohort	Rectus abdominus repair	6.6 years	88% no pain at four months	Three to four months(96%)	96%
Steele et al. [15]	52	R(19-41)	Retrospective cohort	Rectus abdominus repair	12 months	80.7% no pain at four months	Four months (77%)	76.90%									
									Meirhaeghe et al. [16]	33	28.8	Retrospective cohort	Rectus abdominus repair	3 years	81.8% no pain at four months	Two months (81.8%)	90.90%
Muschaweck et al. [11]	89	29	Prospective cohort	Rectus abdominus repair	1 months	79.1% no pain at one month	One month (98%)	83.70%									
									Ziprin et al. [17]	17	27	Prospective cohort	TEP mesh repair	6 months	94% no pain at six months	Six weeks (94%)	94%
Paajanen et al. [7]	41	27	Prospective cohort	TEP mesh repair	4.2 years	95% no pain at 12 months	One month (93%)	95%									
									Paajanen et al. [12]	30	32	Randomized control trial	TEP mesh repair	12 months	90% no pain at three months	Three months (90%)	97%
Piozzi et al. [13]	198	24	Retrospective cohort	TEP mesh repair	12 months	94% no pain at one month	One month (94%)	98.50%									
									Roos et al. [18]	32	22	Prospective cohort	TEP mesh repair	19 months	56% no pain at 12 months	Three months (60%)	72.00%
Kajetanek et al. [2]	27	26.4	Retrospective cohort	Bassini w/ adductor tenotomy	2.75 years	VAS pain 1.0 (0-5)	Four months (92.6%)	92.60%									
									Pokorny et al. [14]	30	25	Prospective cohort	TEP mesh repair	12 months	85% no pain at 12 months	Six weeks (70%)	85%
Van Der Donckt et al. [3]	41	27	Prospective cohort	Bassini w/ adductor tenotomy	12.5 years	78.0% no pain at six months	Seven months (100%)	90.20%									

From the results of this systematic review, it appears that both methods of surgical intervention are very successful procedures, with most having a success rate of above 90%. Return to sport is also around three to six months for most of the studies included, with some as low as one month. Unfortunately, return to sport is not a well-defined term in many of the studies. Some studies only considered return to sport when the athlete was able to fully participate at the competition level. Other studies included when athletes began training again after surgery such as participation in running and cutting, but no full participation in practice or games. When possible, the former definition of return to sport was used for the compilation of Table 1. For other studies, it was unclear which definition was used to determine return to sport time, so the value provided was the value placed in Table 2. Pain scoring was relatively consistent across all of the studies. One study, Kajetanek et al., did not provide time to no pain after operation but used a VAS pain score [2]. A VAS pain score of 1.0 out of 5 is low and indicates minimal pain, which was interpreted as “moderately successful” as defined by Meyers et al. Most of the studies included had pain scores of 90% of patients having no pain at six months, which coincides well with the success rate scores. There was very little difference in pain scores for either treatment although one TEP repair study had a pain score of 56% of patients reporting no pain at 12 months [18]. This was significantly lower than the other studies included.

Overall, from the data collected, both procedures have very high success rates and are almost indistinguishable from each other with the parameters used. This provides difficulty in assessing which procedure should be considered standard of care. When considering that the approaches are based upon the supposed mechanism of injury and underlying pathology and are starkly different procedures, it is surprising to see that almost regardless of treatment, patients have similar outcomes. This systematic review cannot account for the placebo effect as none of the articles used a placebo control, and it is unlikely that any will in the future as the success of both procedures is well documented. In addition, because these procedures are treating different pathologies, it calls into question the name of what is being treated. The terms "sportsman's hernia" and "inguinal disruption" have implications that the pathology involves a weakness in the abdominal wall and involves the underlying viscera [5]. Athletic pubalgia is more encompassing of pain surrounding the pubic bone, which can refer to many variations of the pathology [5]. However, all three terms and others such as Gilmore's groin are all used interchangeably to refer to this injury and this also causes confusion. Thus, it is important not only from a treatment standpoint but also a nomenclature standpoint that we understand exactly what is being treated and if it is effective.

Due to the nature of orthopedics, there are few randomized control trials in this area and only one met the criteria to be included in this study. To differentiate the two procedures further, randomized control trials comparing the two may be an avenue for future research on the subject. Currently, there are no standard methods of assessing pain, return to sport, or success rate as we have defined it, which makes it difficult to compare studies on a similar scale. Standardizing time to no pain after operation would be a great start, for example, six months. If every study included follow-up to six months, it would be very easy to compare the percentage of athletes with no pain. Return to sport also needs to be well-defined because currently, each study has its own definition, which makes comparison difficult. Standardization to a full return to competitive play, not necessarily at the same level, would be another improvement. Success rate is the only measure across all studies that is comparable on a similar scale and there are no significant differences between the two procedures in terms of success rate. Currently, these issues are limitations in this study as we cannot accurately assess differences on the same scale. At best, we can give general results across studies. To compare further and likely discern which procedure most adequately treats athletic pubalgia, a randomized control trial with more specific measures, more frequent follow-up, and standardized clinical outcomes would produce the most noticeable differences between the two treatments. We would recommend using the VAS pain scale measured at consistent intervals for at least six months along with the percentage of athletes with no pain at six months. In addition, we would recommend utilizing more functional outcomes for comparison such as strength, range of motion, balance, and flexibility. These more specific markers could be assessed both preoperatively and postoperatively to assess both changes in function from before to after surgery and compare the two procedures on a more specific and similar scale.

This proposition would hopefully alleviate discrepancies between the proposed pathology and the preferred treatment. However, organizing a randomized control trial such as this could prove difficult as most surgeons performing operations for athletic pubalgia have a personal preference toward one procedure or the other and likely have little experience with the non-preferred procedure. This would make controlling for skill level difficult for one surgeon performing both operations. The solution of course would be to have each surgeon perform the preferred operation, but this results in more of a competition than a randomized control trial. In addition, much of the success of these operations is dependent on physical therapy which may not be consistent across multiple sites. Nonetheless, athletic pubalgia remains a territory for exploration in the future, and studies such as the one proposed would have significant value to the orthopedic community.

This study was very narrow in scope and is thus limited by the targeted procedures. Although they comprise the bulk of the performed operations for athletic pubalgia, there are several others not included in this study that may perform as well or better than the two included. A second limitation of this study is that because both of the screeners worked to extract abstracts from the databases, they were not blinded to the authorship of the studies, which could possibly introduce bias in the screening process. However, to mitigate this, both screeners worked independently and fortunately had substantial agreement. When disagreements occurred, the article in question was fully reviewed except for one article which both reviewers agreed to exclude. All of the articles in question were not included in the final 13 articles and the reviewers were in 100% agreement after an independent review of the full-text articles. Lastly, most of the articles included have lower levels of evidence (2-3), which reduces the overall level of evidence of this systematic review to level 3. There are very few randomized control trials in this area, which lowers the level of evidence, and this is also why further research in the form of randomized control trials is necessary.

## Conclusions

The reported results for the systematic review comparing rectus abdominus repair and posterior wall reinforcement were essentially equivalent. Success rates ranged from about 70% to 90% for both procedures. Due to the lack of standardization of clinical outcomes, it is difficult to discern which procedure is truly better. Future research in this area needs to be conducted to focus on more specific outcomes, more frequent follow-up, and standardization of outcome measures.
